# Supplementary material for: A systematic CRISPR screen reveals an IL-20/IL20RA-mediated immune crosstalk to prevent the ovarian cancer metastasis
Source: eLife. 2021 Jun 11;10:e66222. doi: 10.7554/eLife.66222 (PMC8195602; doi:10.7554/eLife.66222)
Supplement: Supplementary file 1. — The primers used for qRT-PCR and ChIP- qPCR analyses. [file elife-66222-supp1.docx]

**Supplementary File 1.** Primes for qPCR

| Primer | Forward Sequence (5 ’- 3’) | Reverse Sequence (5 ’- 3’) |
| --- | --- | --- |
| Mouse |  |  |
| IL20RA | CACCAGAGGTTGCCCTGACA | TGGTGACACACTGGGACCAC |
| β-actin | GGCTGTATTCCCCTCCATCG | GCACAGGGTGCTCCTCAG |
| NOS2 | ACATCGACCCGTCCACAGTAT | CAGAGGGGTAGGCTTGTCTC |
| IFNAR1 | TTCTGCTCTGACCACCTCCC | ACGAAATGCGAAATCATGTGC |
| CCR2 | ATCCACGGCATACTATCAACATC | CAAGGCTCACCATCATCGTAG |
| TNFα | TTCTGTCTACTGAACTTCGGGGTGATCGGTCC | GTATGAGATAGCAAATCGGCTGACGGTGTGGG |
| ARG1 | CTCCAAGCCAAAGTCCTTAGAG | AGGAGCTGTCATTAGGGACATC |
| IL10 | TTTGAATTCCCTGGGTGAGAA | CTCCACTGCCTTGCTCTTATTTTC |
| CD163 | GGTGGACACAGAATGGTTCTTC | CCAGGAGCGTTAGTGACAGC |
| Ly6G/C | GACTTCCTGCAACACAACTACC | ACAGCATTACCAGTGATCTCAGT |
| CX3CR1 | ACGAAATGCGAAATCATGTGC | CTGTGTCGTCTCCAGGACAA |
| CD68 | GGCCGTTACTCTCCTGCCAT | CTTGGACTAGGCGAGGGTGG |
| IL19 | AGGAGACACCAGGAGAGTCTAGG | AGCAGGTTGTTGGTCATGCAG |
| IL20 | TGGACTGTTCTCCGCTGTGG | TGTATCTTCAGCTTGCACACTATCC |
| IL24 | GCTGTTCCGCAGAGCATTCA | GTCGGAGCATCCAAAGGTGC |
| OAS1A | CCAGCAAGCCTGATCCCAGA | ACAGTGAGCAACTCTAGGGCG |
| IL18 | TGCTGCCTGCATCACACAAAG | GCTGTGCCTGGATGCTTGTAAA |
| IL20RB | CCACCCAGGATGGAGGTCAC | AGAGCTAGTGCCAGCGGAAG |
| Oas1a promotor | |  |
| Site 1 | TCCACTGCCCAATCGAAGGCTC | GCAGTGGATTGCCTTGGTGCTG |
| Site 2 | TCAGACTTCAAAGAACCTGGTCA | TGAGTCCGTGCTCCATGCTA |
| Site 3 | CAGCACCACAGGGGAAAATG | GTGGCTTCCAGCTCCTGAA |
| Il18 promotor | |  |
| Site 1 | GCTTCAGAACACAATACATAAGCC | CTCATCTGACCCCCTAGGCA |
| Site 2 | TGGCAAGTGTCCTAACCAACT | AAACCAAAAACAACGCCCCTC |
| Site 3 | GAACAGCAGCCAGCGTCCTTA | AGCTCATCTCCCAAGCCAAGAAT |
| Human |  |  |
| IL20RA | GCTTCGCTGCGACTCAGAC | CGATCACTGGGCTCCACATCA |
| β-actin | CGTCACCAACTGGGACGA | ATGGGGGAGGGCATACC |
| NOS2 | TTCAGTATCACAACCTCAGCAAG | TGGACCTGCAAGTTAAAATCCC |
| IL1β | AAGCTGAGGAAGATGCTG | ATCTACACTCTCCAGCTG |
| TNFα | GAGGCCAAGCCCTGGTATG | CGGGCCGATTGATCTCAGC |
| ARG1 | GGTTTTTGTTGTTGCGGTGTTC | CTGGGATACTGATGGTGGGATGT |
| IL10 | GACTTTAAGGGTTACCTGGGTTG | TCACATGCGCCTTGATGTCTG |
| CD163 | TTTGTCAACTTGAGTCCCTTCAC | TCCCGCTACACTTGTTTTCAC |
| CD206 | GGGTTGCTATCACTCTCTATGC | TTTCTTGTCTGTTGCCGTAGTT |
| CD68 | CTTCTCTCATTCCCCTATGGACA | GAAGGACACATTGTACTCCACC |
